# HIV-1 Tat protein exposure alters the morphological characteristics and gene expression in the primary mouse cortex endothelial cells and human brain microvascular endothelial cells

**DOI:** 10.21203/rs.3.rs-7736511/v1

**Published:** 2025-10-20

**Authors:** Lili Quan, Ichiro Manabe, Rieko Muramatsu, Jun Zhu

**Affiliations:** National Center of Neurology and Psychiatry; Chiba University; National Center of Neurology and Psychiatry; AUGUSTA UNIVERSITY

**Keywords:** blood-brain barrier, endothelial cell, HIV, Tat, phalloidin, gene, inflammation

## Abstract

HIV-1-associated neurocognitive disorders (HAND) are highly prevalent in the era of combination of antiretroviral therapies. Recent studies suggest that damage of blood-brain barrier (BBB) may serve as an early biomarker of cognitive dysfunction in people living with HIV. This is due to the ability of HIV-1, along with infected monocytes and macrophages, to traverse the BBB via either paracellular or transcellular way. HIV-1 viral proteins have been shown to disrupt tight junctions within the BBB, thereby directly compromising its structural and functional integrity. This study determined the effects of the HIV-1 transactivator of transcription (Tat) protein on the morphological profiles and gene expression of mouse prefrontal cortex endothelial cells (ECs) and human brain microvascular endothelial cells (HBMVEC). Both mouse ECs and HBMVEC were exposed *in vitro* to 12.5 nM recombinant Tat1 – 86 for 48 hours. After treatment, cells were immunostained with CD31, anti-Tat, DAPI or phalloidin, and harvested for RNA sequencing to access changes in gene expression. Staining results showed a reduction in CD31 expression accompanied by an increase in phalloidin staining intensity in both mouse ECs and HBMVECs after 48-hour Tat exposure. Moreover, the phalloidin staining revealed disruption of actin cytoskeleton structure in both mouse ECs and HBMVECs after 48-hour Tat exposure. RNA sequencing analysis of mouse ECs and HBMVECs exposed to Tat displayed strikingly comparable transcriptomic signatures, as confirmed by gene set enrichment analysis (GSEA). In particular, both mouse ECs and HBMVECs showed significant upregulation of hallmark inflammatory response pathways following 48-hour Tat exposure. These findings provide mechanistic insight into HIV-1 Tat drives endothelial injury, leading to both morphological and transcriptional alterations.

## Background

People living with HIV (PLWH) are at increased risk of neurocognitive impairments, including depression, and tend to display higher levels of impulsive behavior, particularly in the context of substance abuse. The lifetime prevalence of cognitive depression among PLWH is estimated to be nearly twice that HIV-seronegative individuals. (Ickovics et al., 2001, Mao et al., 2008, Rubin and Maki, 2019). In the United States, approximately 39% of HIV-infected individuals are reported to experience depression (Bhatia and Munjal, 2014, Campos et al., 2010, Castellon et al., 1998, Rabkin, 2008, Savetsky et al., 2001, Too et al., 2021). Despite the widespread use of combined antiretroviral therapies (cART), approximately 39.6% of HIV-1 seropositive individuals continue to experience asymptomatic to mild symptoms of neurocognitive impairment (Keng et al., 2023). While cART effectively suppresses acute viral replication, accumulating evidence suggests that persistent exposure to HIV-1 viral proteins within the CNS contributes to neurocognitive vulnerability by promoting the elevation of proinflammatory cytokines. Elucidating how these viral proteins drive pathophysiological cascades that culminate in cognitive impairment in PLWH will refine mechanistic understanding and guide preventive interventions for the comorbidity of HIV-1–associated depression and cocaine use. The HIV-1 trans-activator of transcription (Tat) protein regulates viral promoter activity by binding to the promoter and recruiting the transcriptional machinery, thereby driving a robust increase in viral transcript production. Tat is produced and secreted by HIV-1–infected cells, including those within viral reservoirs that persist despite cART. Notably, Tat remains detectable in the cerebrospinal fluid and blood of approximately 40–50% of individuals on cART (Johnson et al., 2013, Mediouni et al., 2012).

The blood–brain barrier (BBB) plays a critical role in regulating the transport of oxygen and essential nutrients into the central nervous system (CNS) while protecting the brain by preventing the entry of harmful substances (Alahmari, 2021, Kadry et al., 2020). Endothelial cells (ECs) form the foundation of the BBB, which is composed of brain vascular ECs supported by a basement membrane on the abluminal side and surrounded by pericytes and astrocytic endfeet. In concerts with tight junction proteins, ECs regulate the protective and barrier functions of the BBB (Kadry, Noorani, 2020, Segarra et al., 2021). In the early stages of HIV infection, exposure to virions and viral proteins causes endothelial injury and disrupts BBB integrity, thereby facilitating viral entry into the brain and productive infection of resident CNS cells, particularly microglia (Borrajo et al., 2021, Ginsberg et al., 2018). In addition, the BBB also serves as a critical entry site for HIV-infected monocytes and macrophages, which can cross the barrier through either paracellular or transcellular pathways (Gonzalez-Scarano and Martin-Garcia, 2005, Persidsky and Poluektova, 2006). In general, virions and viral proteins that cross the BBB trigger increased inflammation and oxidative stress, resulting in neuronal injury and subsequent neurodegeneration (Kaul et al., 2005, Mattson et al., 2005). Consequently, BBB disruption has been identified as an early biomarker of cognitive dysfunction (Nation et al., 2019). Nevertheless, although nearly 50% of HIV-1–infected patients on cART exhibit neuroinflammation and anti-inflammatory agents have been shown to block HIV-1 reactivation from latency, the contribution of the BBB to the neuropathogenesis of HAND remains largely unexplored.

HIV-1 Tat protein is the first HIV-1 protein proven to rigorously affect ECs in the pre-ART era, promoting vascular endothelial dysfunction (Chandran et al., 2025). Previous studies have shown that exposure of brain ECs to HIV-1 Tat induces cytoskeletal changes, disrupts efflux transporter function, and compromises tight junction barrier function (Andras et al., 2003, Avraham et al., 2004, Xu et al., 2012). Tat-induced ECs injury also elevates activator protein-1 as well as proinflammatory cytokines and chemokines. In this study, mouse cortical ECs were isolated and exposed to recombinant Tat_1 – 86_ (rTat_1 – 86_) protein, with human brain microvascular endothelial cells (HBMVECs) included for comparison. Tat-induced morphological changes were subsequently assessed via immunohistochemistry with CD31 and Rhodamine Phalloidin. Additionally, the transcriptomic profiles of mouse ECs and HBMVECs exposed to rTat_1 – 86_ were analyzed using RNA-sequencing. These results provide insights into the role of ECs and their associated gene expression in neuropathogenesis, thereby contributing to a better understanding of HIV-1–associated neurocognitive disorders (HAND).

## Materials and methods

### Animals

Female C57BL/6J mice with an age of 3 weeks were obtained from Japan SLC (Japan) for use in this study. All experiments were conducted upon animal arrival. All experimental procedures complied with protocols approved by the Institutional Animal Care and Use Committee (IACUC) at National Center of Neurology and Psychiatry.

### Materials

Dulbecco’s Modified Eagle’s Medium (DMEM), Bovine Serum Albumin (BSA), Dulbecco’s Modified Eagle’s Medium/Nutrient Mixture F-12 Ham (DF12), Sodium bicarbonate, Insulin, apo-Transferrin human, Puromycin dihydrochloride, Protease Inhibitor P8340, and Basic fibroblast growth factor (bFGF) were purchased from Sigma-Aldrich (St. Louis, MO). Heat inactivated fetal bovine serum (FBS) and trypsin/EDTA were purchased from Corning Life Sciences (Woodland, CA). Penicillin/streptomycin was purchased from Gibco Life Technologies Corporation (Grand Island, NY). The recombinant HIV-1 transactivator of transcription (rTat_1 – 86_) was purchased from ImmunoDX (Woburn, MA).

### Isolation of endothelial cells of mouse prefrontal cortex

Mouse prefrontal cortex ECs of three-week-old C57BL/6J female mice were isolated as described in the previous publication (Muramatsu et al., 2012). In brief, for an individual experiment, mice were under deep anesthesia by ice-induced hypothermia and prefrontal cortex tissues were dissected on ice-cold dish and cut into small pieces as much as possible. The brain tissues were then incubated in Digestion solution [1:1 Collagenase type (Worthington) / Dulbecco’s Modified Eagle’s Medium (DMEM, Sigma-Aldrich) at 1 mg/ml as final concentration] at 37°C, at 85 min after incubation, DNase-I (Sigma) was added into to the Digestion solution containing tissues (10 mg/mL, final concentration) for additional 5 min. After a total of 90 min incubation, the solution was centrifuged at 450g for 5 min. Following the first centrifugation, the supernatant was discarded, and the pellet (P1) was suspended in 20 mL DMEM and subjected to second centrifugation (1000g for 10 min). The supernatant was discarded and the pellet (P2, large but soft) was resuspended in 20 mL DMEM and subjected to third centrifugation (1000g for 10 min). To achieve ECs, the pellet (P3) was suspended in 20 mL DMEM containing 20% BSA (Sigma) and subjected to fourth centrifugation (1000g for 10 min). After the 4th centrifugation, the upper and middle layers of supernatant were discarded, and pellet (P4) was suspended in 20 mL DMEM containing Collagenase/Dispase (Roche, 1 mg/mL, final concentration) followed by 45 min-incubation at 37°C. At 40 min after the incubation, DNase-I (Sigma) was added into to the Collagenase/Dispase in DMEM solution containing tissues (10 mg/mL, final concentration) for additional 5 min followed by the fifth centrifugation (1000g for 10 min). The supernatant was discarded, and the supernatant was discarded, and the pellet (P5) was resuspended in 20 mL DMEM and subjected to sixth centrifugation (1000g for 10 min). While the resuspensions and centrifugations, Percoll solution containing 5.2 mL Percoll (GE Healthcare), 1xPBS 9.8 mL, 520 μL 10xPBS and 520 μL FBS, total 16 mL) was centrifuged at 30,000g at 4°C for 60 min. After 60 min centrifugation, the Percoll solution was separated into three layers, in which the top layer of solution was saved for the next step. After the sixth centrifugation, the supernatant was discarded, and the pellet (P6) was resuspended in 1 mL DMEM and transferred to the saved top layer of Percoll solution and subjected to seventh centrifugation (1000g for 10 min) at 4°C. Following the seventh centrifugation, three layers of solution were observed, in which the middle layer of solution (containing ECs) was aspirated using 10 mL syringe and transferred to 30 mL DMEM followed by last centrifugation (1000g for 10 min). After the centrifugation, the supernatant was discarded, and was discarded, and the pellet (P7) was resuspended in culture medium [DF12 (120 mg/mL), NaHCO_3_ (12 mg/mL), 1% Penicillin/Streptomycin, 10% FBS, Heparin (5 μg/mL), Insulin (5 μg/mL), Sodium selenite (5 ng/mL), 100 nM Hydrocortisone, Apo-transferrin (5 μg/mL), bFGF (1 μg/mL), and Puromycin (1 μg/mL), pH 7.2] followed by culture in a 5% CO_2_ incubator at 37 °C. Twenty-four hours after incubation, half medium was replaced with fresh medium, and ECs were cultured for additional 10 days until assay.

### In vitro exposure of mouse ECs and HBMVECs to recombinant Tat

HBMVECs were purchased from iXCells (SKU: 10HU-051) and cultured in endothelial cell growth medium (MD-0010; iXCells) according to the manufacturer’s instructions. Mouse ECs were cultured in the medium described above. Both types of cells HBMVECs were maintained at 37°C in a 5% CO_2_ incubator for 10 days before Tat exposure. For *in vitro* Tat exposure, cells grew to 80–90% confluence on 24-well plates which then were seeded at a density of 10,000 cells/well in 96-well plates. For individual experiment, triplicate-well cells were exposed to control medium containing 0.1% protease inhibitor cocktail for preventing Tat protein degradation or the same medium containing recombinant Tat_1 – 86_ or heated Tat (95°C for 5 min) at a 12.5 nM final concentration and incubated at 37°C, 5% CO_2_ incubator for the designed time. For all data presented in this study, 48 hours after Tat treatment, cells were harvested for fixation for immunocytochemical analysis and RNA isolation.

### Immunocytochemistry analysis

Cells were fixed with 4% paraformaldehyde (PFA) in phosphate buffer (PB) for 15 min at room temperature, then rinsed twice with PBS and permeabilized twice with 0.1% Triton X-100 in PBS for 10 min. Cells were then blocked with 3% normal donkey serum (NDS, Sigma Aldrich) for 1 h at room temperature and incubated with the following primary antibodies in blocking solution: rat anti-CD31 (1:500, 557355; BD Pharmingen), rabbit anti-CD31 (1:500, ab182981; Abcam), and rhodamine phalloidin (1:400, R415; Thermo Fisher Scientific) at 4°C overnight. After rinsing twice with PBS, sections were incubated with Alexa 488-bound secondary antibody (1:500; Thermo Fisher Scientific) and 4’,6-diamidino-2-phenylindole (DAPI, 1 mg/mL; 1:2000, Dojindo Laboratories) for 1 h at room temperature.

### Acquisition and analysis of the fluorescence intensity and images

Fluorescence images of each plate were generated using In cell analyzer 2000 or Olympus Fluoview FV 3000. To measure the fluorescence intensity, the acquired images were quantified using Fuji/ImageJ software. Triplicate samples (ECs in three wells) were performed for each experiment for either control or Tat exposure. Three images from different sites per well were randomly taken for data analysis. Data were expressed as mean ± SE of fluorescence intensity.

### RNA isolation

Total RNAs were isolated from HIV-1 Tat-treated mouse ECs or HBMVECs using TRIzol reagent (Thermo Fisher Scientific) and purified using the RNeasy Micro Kit (Qiagen) according to the manufacturer’s instructions.

### RNA-seq analysis

Total RNAs from Tat-treated mouse ECs or HBMVECs were described above. For RNA-seq analysis from mouse ECs, library preparation was performed using SMART-Seq HT Kit (Takara) and Nextera XT DNA Library Preparation Kit (Illumina). Whole transcriptome sequencing was performed in 100-base single-ended mode on the Illumina NovaSeq 6000 platform (Illumina). Bcl2fastq conversion software version 2.20 (Illumina) was used for base calling. Sequencing reads were mapped to the mouse reference genome sequence (mm10) using Hista2 version 2.2.1, SAMtools version 1.3.1, and Stringtie version 2.1.7. The number of fragments per kilobase of exon per million mapped fragments (FPKMs) was calculated using Cufflinks version 2.2.1. For RNA-seq analysis from HBMVECs treated with rTat_1 – 86_ for 8 hours at 37°C, 5% CO_2_ incubator, poly-A mRNA was isolated from total RNA using Oligo-dT beads from a NEBNext Poly(A) RNA Magnetic Isolation Module (NEB), and RNA-seq libraries were constructed using a NEBNext Ultra II RNA Library Prep Kit from Illumina (NEB) according to manufacturer instructions. Libraries were sequenced either single-end or paired-end using an Illumina NovaSeq sequencer. The reads were aligned with the hg38 human genome using STAR (Dobin et al., 2013). HOMER (Heinz et al., 2010) was used to evaluate the aligned read files and expression analysis on RNA-seq data. Differential gene expression analyses were performed using DESeq2 (v1.42.0) (Love et al., 2014) and Bioconductor (v3.18) in R (v4.3.2). The RNA-seq data were presented from three independent experiments. All original data of RNA-sequencing were stored in NCBI Gene Expression Omnibus (GEO) database with ID: GSE307483 for mouse ECs and GSE307481 for HBMVECs). Gene set enrichment analysis (GSEA) was performed using GSEA(Subramanian et al. 2005) software version 4.3.3, with rank files generated from expression data processed using DESeq2. Canonical pathway enrichment analysis was performed using Ingenuity Pathway Analysis (IPA) software (Qiagen). For mouse ECs, a threshold of p < 0.1 was applied to define significantly abundant genes. Z-score was calculated to determine the activation status of relative pathways, and those canonical pathways with −LogP > 5.0 and Z-score >|3.0| were considered as significantly activated or inhibited. For HBMVECs, pathways with −LogP > 1.3 and Z-score >|1.0| were considered significantly regulated.

### Statistical Analysis

Results are presented as mean ± SEM. The number of n represents the number of independent experiments from each experiment group. The fluorescence intensity involving comparisons between controls and Tat-treated samples, unpaired Student’s *t* tests were used to determine any difference between Tat-treated and untreated samples using GraphPad version 10.3.1. For multiple comparisons, one-way ANOVA followed by Tukey’s post hoc test was applied. *p* < 0.05 were considered significant.

## Results

### Effects of in vitro rTat_1 – 86_ exposure on CD31 fluorescence intensity of mouse ECs and HBMVECs

**Figure 1:**
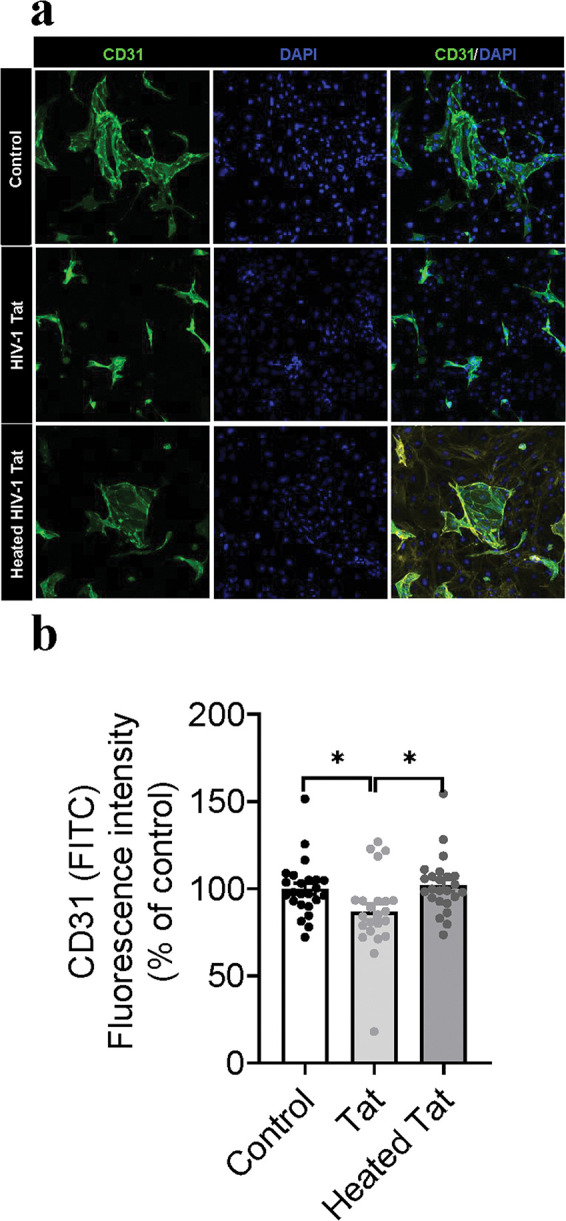
HIV-1 Tat reduces expression of adhesion molecule CD31 in the mouse ECs. (A) Representative images of the endothelial cell marker CD31 (green). Cell nuclei were stained with DAPI (blue). The mouse ECs were treated with medium control, rTat_1–86_ (12.5 nM), or heated rTat_1–86_ (12.5 nM) for 48 hours. (B) Quantification of CD31 fluorescence intensity. Data of rTat_1–86_-trested EVs are represented as a percentage relative to the controls with medium or heated rTat_1–86_. Data are presented as mean ± SEM of three independent experiments. *p* values were calculated using one-way ANOVA post Turkey’s test and are shown.

**Figure 2:**
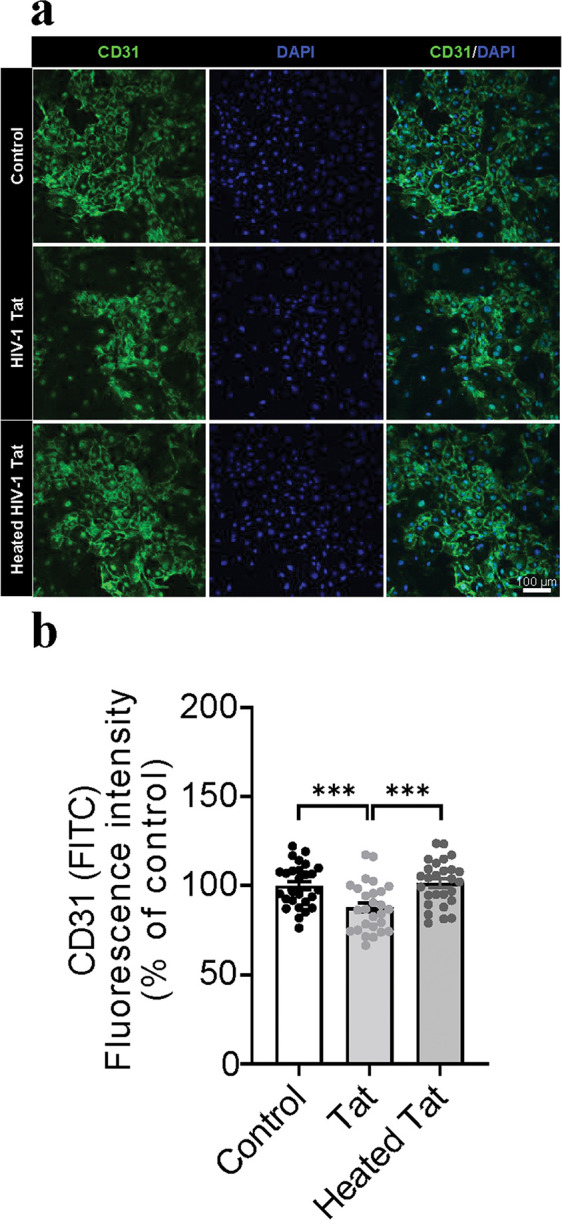
HIV-1 Tat reduces expression of adhesion molecule CD31 in HBMVECs. (A) Representative images of the endothelial cell marker CD31 in HBMVECs (green). Cell nuclei were stained with DAPI (blue). HBMVECs were treated with control, rTat_1–86_ (12.5 nM), or heated rTat_1–86_ (12.5 nM) for 48 hours. (B) Quantification of CD31 fluorescence intensity. Data of rTat_1–86_-trested HBMVECs are represented as a percentage relative to the controls with medium or heated rTat_1–86_. Data are presented as mean ± SEM of three independent experiments. *p* values were calculated using one-way ANOVA post Turkey’s test and are shown.

To determine whether HIV-1 Tat protein changes morphological profiles of the ECs, immunostaining with CD31 in both mouse ECs and HBMVECs was conducted after 6, 12, 24, 48 h rTat_1 – 86_ exposure. Figure 1A shows the representative images of CD31 fluorescence intensity of mouse ECs with 48-h exposure to medium control, 12.5 nM rTat_1 – 86_ or heated rTat_1 – 86_. One-way ANOVA reveals that a significant change in the CD31 intensity among treatment with rTat_1 – 86_, heated rTat_1 – 86_ and control (F_(2, 68)_ = 4.6, *p* = 0.013). Compared to medium control (100 ± 3.3) or heated rTat_1 – 86_ (102 ± 3.3), Tat treatment significantly reduced CD31 fluorescence intensity of mouse ECs by 13.3% (Tat exposure, 86.8 ± 4.8, *t*_(45)_ = 2.3, *p* = 0.027, Fig. 1B). Figure 2A shows the representative images of CD31 fluorescence intensity of HBMVECs with 48-h exposure to medium control or 12.5 nM rTat_1 – 86_ or heated rTat_1 – 86_. Compared to controls with medium (100 ± 2.3) or heated rTat_1 – 86_ (101 ± 2.4), 48-h rTat_1 – 86_ exposure significantly alters the CD31 fluorescence intensity of HBMVECs (one-way ANOVA, F_(2,78)_ = 9.17, *p* = 0.0003). No difference in the CD31 fluorescence intensity was found between control with medium (100 ± 2.3) and heated Tat exposure (101 ± 2.4, t_(52)_ = 0.41, *p* > 0.05), whereas rTat_1 – 86_ exposure induced a 12.1% reduction of the CD31 fluorescence intensity (87.8 ± 2.6) relative to either control with heated Tat_1 – 86_ (t_(52)_ = 3.79, *p* = 0.0004) or control with medium (t_(52)_ = 3.47, *p* = 0.001, Fig. 2B).

### Effects of in vitro rTat_1 – 86_ exposure on phalloidin fluorescence intensity of mouse ECs and HBMVECs

**Figure 3:**
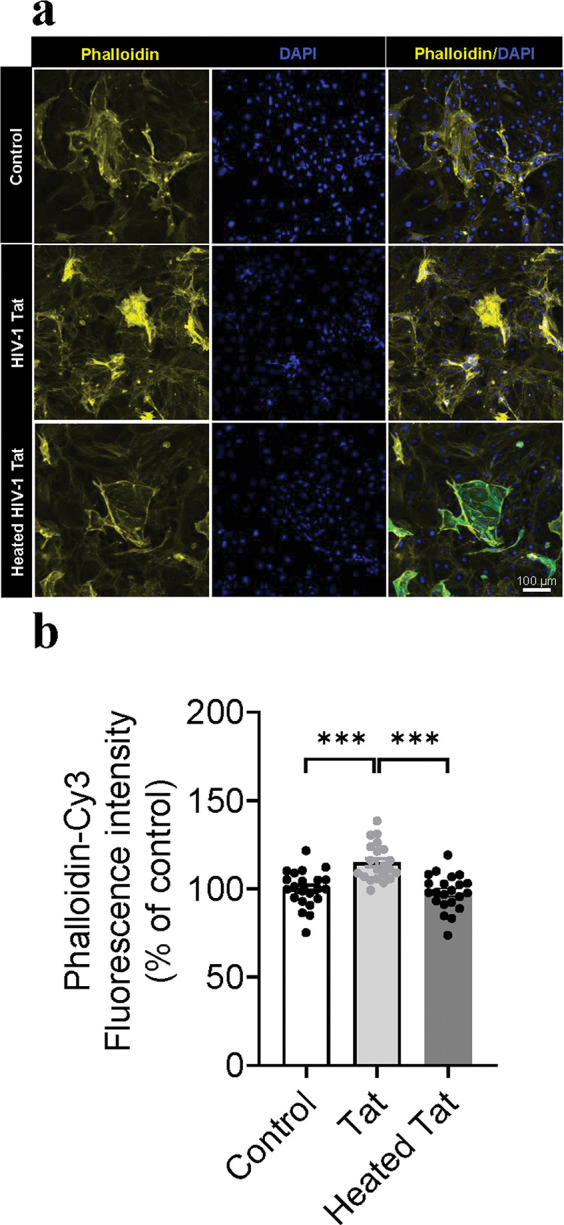
HIV-1 Tat alters actin filament arrangement in the mouse ECs. (A) Representative images of the actin filament staining using rhodamine phalloidin (yellow). Cell nuclei were stained with DAPI (blue). The mouse ECs were treated with medium control, rTat_1–86_ (12.5 nM), or heated rTat_1–86_ (12.5 nM) for 48 hours. (B) Quantification of rhodamine phalloidin fluorescence intensity. Data of rTat_1–86_-trested EVs are represented as a percentage relative to the controls with medium or heated rTat_1–86_. Data are presented as mean ± SEM of three independent experiments. *p* values were calculated using one-way ANOVA post Turkey’s test and are shown.

**Figure 4:**
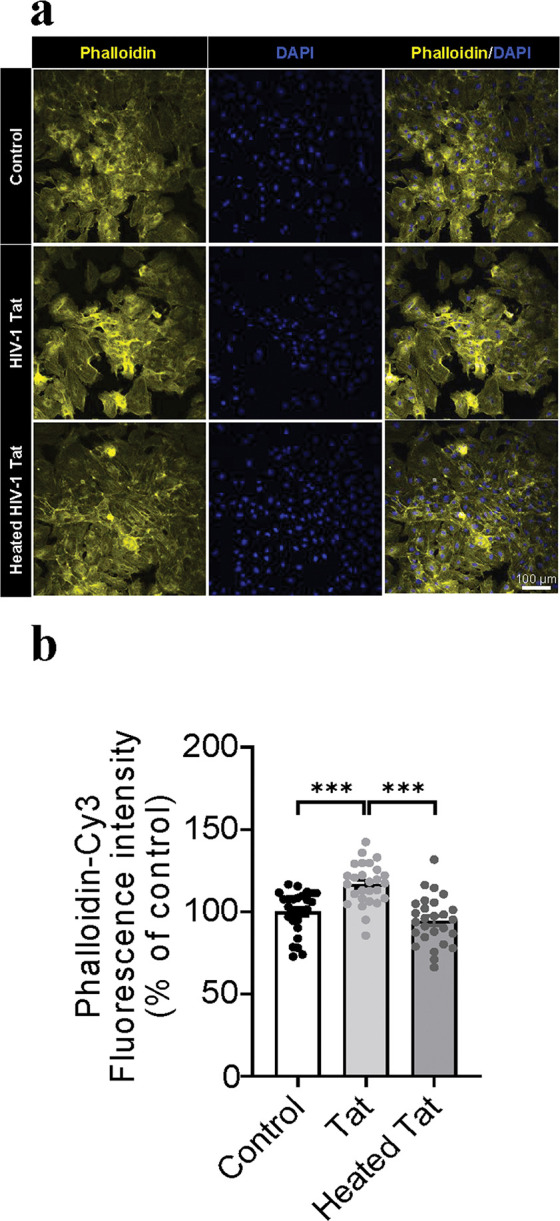
HIV-1 Tat alters actin filament arrangement in HBMVECs. (A) Representative images of actin filament staining in HBMVECs using rhodamine phalloidin (yellow). Cell nuclei were stained with DAPI (blue). HBMVECs were treated with control, rTat_1–86_ (12.5 nM), or heated rTat_1–86_ (12.5 nM) for 48 hours. (B) Quantification of rhodamine phalloidin fluorescence intensity. Data of rTat_1–86_-trested HBMVECs are represented as a percentage relative to the controls with medium or heated rTat_1–86_. Data are presented as mean ± SEM. of three independent experiments. *p* values were calculated using one-way ANOVA post Turkey’s test and are shown.

To determine whether HIV-1 Tat protein disrupts actin cytoskeleton structure of ECs, immunostaining with phalloidin in both mouse ECs and HBMVECs, was performed after 48 h rTat_1 – 86_ exposure. Figure 3A shows the representative images of phalloidin fluorescence intensity of mouse ECs with 48-h exposure to medium control, 12.5 nM rTat_1 – 86_ or heated rTat_1 – 86_. One-way ANOVA reveals a significant alteration of phalloidin fluorescence intensity among controls with medium or heated rTat_1 – 86_ and rTat_1 – 86_ (F_(2, 62)_ = 17.2, *p* < 0.0001). Compared to controls with medium (100 ± 2.2) or heated rTat_1 – 86_ (98 ± 2.2), 48-h rTat_1 – 86_ exposure increased phalloidin fluorescence intensity in Tat-treated mouse ECs (114.9 ± 2.3, t_(41)_ = 4.7, *p* < 0.0001 and t_(41)_ = 5.38, *p* < 0.0001, Fig. 3B). Figure 4A shows the representative images of phalloidin fluorescence intensity of HBMVECs with 48-h exposure to medium control, 12.5 nM rTat_1 – 86_ or heated rTat_1 – 86_. One-way ANOVA shows that Tat exposure alters phalloidin fluorescence intensity (F_(2, 78)_ = 19.7, *p* < 0.0001). Post hoc analysis reveals that 48-h rTat_1 – 86_ exposure increased phalloidin fluorescence intensity by % (116.7 ± 2.4), compared to either medium control (100 ± 2.5, t_(52)_ = 4.8, *p* < 0.0001) or heated rTat_1 – 86_ (94.5 ± 2.9, t_(52)_ = 5.9, *p* < 0.0001), while no difference was found between medium control and heated rTat_1 – 86_, t_(52)_ = 1.4, *p* = 0.1491, Fig. 4B. Moreover, the phalloidin staining showed that actin cytoskeleton structure was disrupted in both mouse EVs and HBMVECs after 48-h Tat exposure.

### Transcriptome profiles of rTat_1 – 86_ exposure on mouse ECs

**Figure 5:**
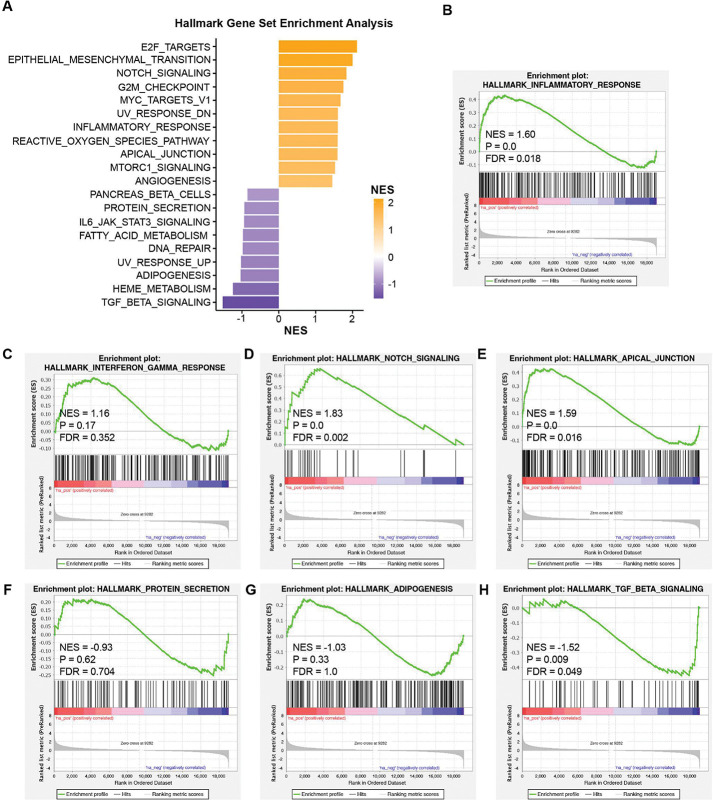
Gene set enrichment analysis (GSEA) of HIV-1 Tat-treated mouse ECs. (A) Top 20 up- or down-regulated gene sets enriched in hallmark pathways with an FDR < 0.15. (B-H) GSEA plots for hallmark gene set of inflammatory response (B), interferon gamma response (C), notch signaling (D), apical junction (E), protein secretion (F), adipogenesis (G), and TGF beta signaling (H).

**Figure 6:**
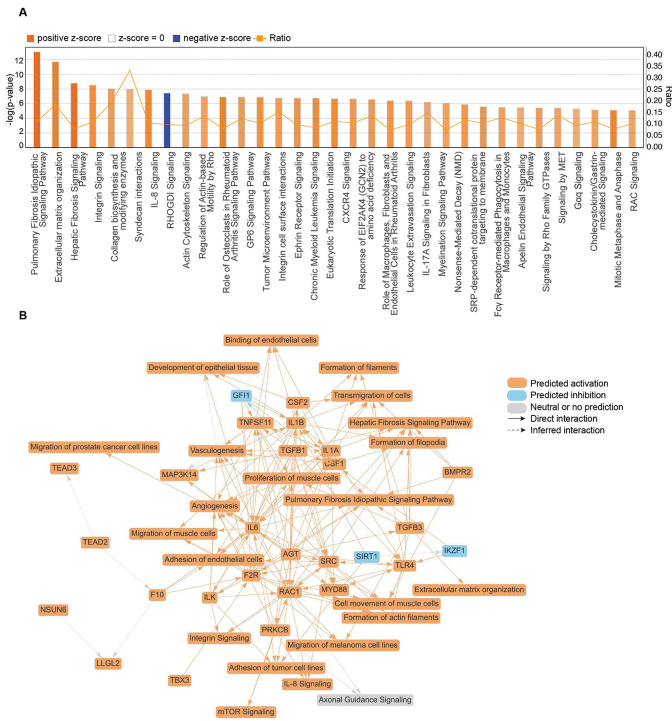
Ingenuity pathway analysis (IPA) identifying significant canonical pathways in HIV-1 Tat-treated mouse ECs. (A) Enriched canonical pathways in the IPA (*p* < 0.1). Orange bars represent activated pathways with positive z-scores, while blue bars represent inhibited pathways with negative z-scores. The ratio (depicted by orange dots connected by an orange line) indicates the proportion of genes from the dataset that map to a given pathway relative to the total number of genes associated with that pathway. (B) Graphical summary of the network diagram in IPA, illustrating the significant regulatory effects after HIV-1 Tat treatment in mouse ECs. Orange nodes indicate predicted activation, blue nodes indicate predicted inhibition, and gray nodes indicate neutral or no prediction.

To further explore the transcriptional regulation of rTat_1 – 86_ on ECs, we performed gene set enrichment analysis (GSEA) using the entire RNA sequence dataset from mouse ECs exposed or unexposed to rTat_1 – 86_ for 48 h. The top 20 enriched pathways in rTat_1 – 86_-treated mouse ECs ranked by normalized enrichment score (FDR < 0.15) are shown in Fig. 5A and Supplementary **Table 1**. Overall, the top enriched pathways in the hallmark analysis indicated modulation of immune response (inflammatory response, IL6/JAK/STAT3 signaling), cell-cell adhesion (apical junction), cell proliferation (E2F targets, G2M checkpoint, Myc targets V1), protein secretion, metabolic regulation (fatty acid metabolism, heme metabolism), and involvement of notch signaling, mTORC1 signaling, and TGF beta signaling were enriched. GSEA plots showed elevated inflammatory response (NES = 1.60, FDR = 0.018), interferon gamma response (NES = 1.16, FDR = 0.352), notch signaling (NES = 1.83, FDR = 0.002), and apical junction (NES = 1.59, FDR = 0.016) (Fig. 5B–E). Whereas downregulation of protein secretion (NES = −0.93, FDR = 0.704), adipogenesis (NES = −1.03, FDR = 1.0), and TGF beta signaling (NES = −1.52, FDR = 0.049) was observed after HIV-1 Tat treatment (Fig. 5F–H). We further explored the pathways of differentially abundant genes (*p* < 0.1) in HIV-1 Tat-treated ECs compared to controls using ingenuity pathway analysis (IPA) (Fig. 6A). Consistent with the observation of elevated phalloidin intensity in Tat-treated mouse ECs, pathway analyses also indicated the activation of extracellular matrix organization (z-score 4.472, *p* = 8.89E-14), integrin signaling (z-score 4.025, *p* = 3.22E-9), collagen biosynthesis and modifying enzymes (z-score 3.606, *p* = 9.23E-9), actin cytoskeleton signaling (z-score 3.5, *p* = 4.73E-8), regulation of actin-based motility by Rho (z-score 3.317, *p* = 1.11E-7), integrin cell surface interactions (z-score 3.606, *p* = 1.78E-7), and signaling by Rho family GTPases (z-score 3.606, *p* = 3.94E-6), which are related to the actin filament organization. Figure 6B shows the most important biological insights gained from IPA, including entities such as typical pathways in integrin signaling, IL-8 signaling, and mTOR signaling; upstream regulators (TGFB1, IL1A, IL1B, IL6, etc.); and biological functions of actin filaments formation, extracellular matrix organization, and endothelial cell adhesion; as well as their connections.

### Transcriptome profiles of rTat_1 – 86_ exposure on HBMVECs

**Figure 7:**
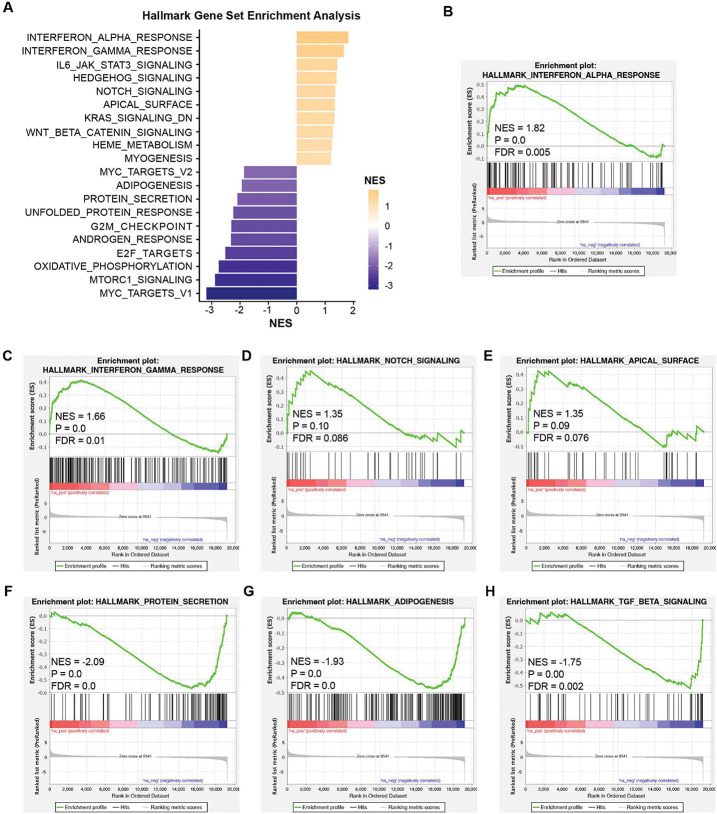
Gene set enrichment analysis (GSEA) of HIV-1 Tat-treated HBMVECs. (A) Top 20 up-or down-regulated gene sets enriched in hallmark pathways with an FDR < 0.15. (B-H) GSEA plots for hallmark gene set of interferon alpha response (B), interferon gamma response (C), notch signaling (D), apical surface (E), protein secretion (F), adipogenesis (G), and TGF beta signaling (H).

**Figure 8:**
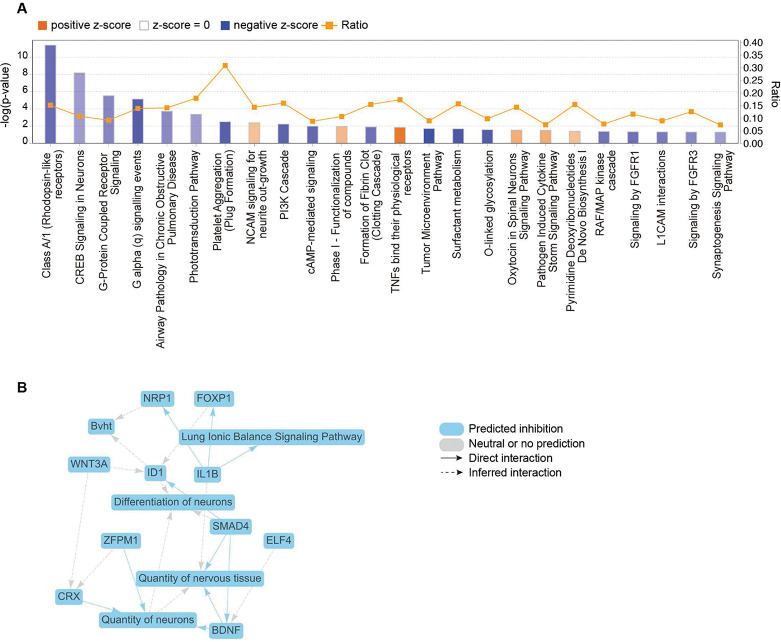
Ingenuity pathway analysis (IPA) identifying significant canonical pathways in HIV-1 Tat-treated HBMVECs. (A) Enriched canonical pathways identified in the IPA. Orange bars represent activated pathways with positive z-scores, while blue bars represent inhibited pathways with negative z-scores. The ratio (depicted by orange dots connected by an orange line) indicates the proportion of genes from the dataset that map to a given pathway relative to the total number of genes associated with that pathway. (B) Graphical summary of network diagram in IPA, illustrating the significant regulatory effect after HIV-1 Tat-treatment in HBMVECs. blue nodes indicate predicted inhibition, and gray nodes indicate neutral or no prediction.

To determine the transcriptional changes in HBMVECs after rTat_1 – 86_ exposure, we also conducted bulk RNA-seq analysis using RNAs isolated from HBMVECs exposed or unexposed to rTat_1 – 86_. Figure 7A presents the top 20 differentially regulated pathways by NES in Tat-treated HBMVECs as indicated by GSEA analysis (FDR < 0.15) (Supplementary Table 2). Similar to mouse ECs, HBMVECs with the exposure of rTat_1 – 86_ exhibited the upregulated enrichment in the hallmark pathways of immune responses, such as interferon alpha response (NES = 1.82, FDR = 0.005), interferon gamma response (NES = 1.66, FDR = 0.01), notch signaling pathway (NES = 1.35, FDR = 0.086), and apical surface (NES = 1.35, FDR = 0.076) (Fig. 7B–E). However, protein secretion (NES = −2.09, FDR = 0.0), adipogenesis (NES = −1.93, FDR = 0.0), and TGF beta signaling (NES = −1.75, FDR = 0.002) are downregulated (Figure F–H). In the top enriched canonical pathways from IPA in HBMVECs (Fig. 8A), pathways related to neuron differentiation and function, such as Class A/1 (Rhodopsin-like receptors) (z-score – 1.697, *p* = 3.99E-12), CREB signaling in neurons (z-score – 1.134, *p* = 6.48E-9), and nervous tissue development and maintenance, such as Neural Cell Adhesion Molecule (NCAM) signaling for neurite out-growth (z-score 1.000, *p* = 4.10E-3), were identified. The graphical summary obtained from IPA highlighted genes such as BDNF, IL1B, and SMAD4, and their downstream effects control the development and maintenance of nervous tissue (Fig. 8B). Thus, it is possible that HIV-1 Tat directly regulates the EC function, leading to neuronal dysfunction and inducing HAND in infected patients.

## Discussions

The goal of the current study is to determine how exposure of brain ECs to HIV-1 Tat protein affects endothelial cell morphology and the expression of genes associated with endothelial cell damage. Hence, we report several novel findings based on immunochemistry and RNA-seq, followed by pathway analysis. First, exposure of mouse ECs or HBMVECs to HIV-1 Tat protein significantly reduced CD31 intensity, also known as platelet endothelial cell adhesion molecule-1 (PECAM-1), suggesting Tat-induced endothelial dysfunction. Second, after 48-hour Tat exposure, phalloidin staining, an indicator of actin filaments, was significantly reduced in both mouse ECs and HBMVECs. Additionally, the actin cytoskeleton structure based on Tat-induced disruption of phalloidin was damaged. Third, intriguingly, RNA sequencing analysis of mouse ECs and HBMVECs exposed to HIV-1 Tat revealed highly comparable transcriptomic signatures, as demonstrated by GSEA. Particularly both mouse ECs and HBMVECs exhibited significant upregulation of hallmark inflammatory response pathways following 48-hour Tat exposure. These findings elucidate the molecular mechanisms underlying HIV-1 Tat-induced endothelial cell damage, which drives both morphological alterations and changes in gene expression

An important finding of the current study is that HIV-1 Tat exposure induces marked morphological alterations in both mouse ECs and HBMVECs, as evidenced by reduced CD31 expression and enhanced phalloidin staining intensity. CD31, a transmembrane protein abundantly expressed at endothelial junctions of the BBB, plays a critical role in maintaining barrier integrity. Dysfunction of CD31 signaling has been implanted in the progression of inflammatory diseases (Cheung et al., 2020, Woodfin et al., 2007). Decreased levels of the protein are closely associated with BBB breakdown, progressive neuronal degeneration, and the onset of cognitive impairment in neurodegenerative patients (Wang et al., 2023, Wimmer et al., 2019). Tat-induced endothelial cell damage may facilitate inflammation by promoting the transmigration of HIV-1–infected monocytes across the BBB (Schechter et al., 2017, Vinhaes et al., 2021). HIV-1 Tat protein has been detected in the circulation at concentrations of approximately 4.5 ng/mL in both HIV-1-positive and HIV-seronegative individuals, and is measurable in both plasma and serum (Shmakova et al., 2024). These levels are consistent with the Tat concentrations (1–5 ng/mg) detected in the brains of HIV-infected individuals (Fan et al., 2016, Westendorp et al., 1995, Xiao et al., 2000). Thus, this study provides novel evidence that HIV-1 Tat protein compromises BBB integrity by reducing CD31 expression upon reaching the barrier. In addition, Tat disrupts the F-actin cytoskeleton in both mouse ECs and HBMVECs, as evidenced by elevated phalloidin staining intensity. Phalloidin, a toxin derived from the mushroom Amanita phalloides, binds specifically to filamentous actin (F-actin), making it an essential tool in cell biology for visualizing and studying the organization of the actin cytoskeleton. During initial HIV infection, HIV-1 virus manipulates the F-actin network of the target cells to promote viral entry and movement toward the nucleus (Marquez et al., 2018). In the current study, Tat may directly bind to actin filaments, while cytoskeletal subversion occurs indirectly through the manipulation of actin regulatory proteins, which are disrupted at multiple levels.

To better understand how HIV-1 Tat impacts brain ECs, transcriptomic analysis demonstrated that Tat exposure significantly enriched pathways related to immune responses, actin filament organization, protein secretion, and neuronal development and function. Gene set enrichment analysis revealed multiple pathways that were significantly enriched in both mouse ECs and HBMVECs following 48-hour exposure to Tat. Particularly, hallmark IL6/JAK/STAT3 signaling pathways were significantly enriched in both Tat-treated mouse ECs and HBMVECs. Given that chronic neuroinflammation plays a central role in HAND pathogenesis (Sreeram et al., 2022, Wojna, 2022), these results provide strong evidence connecting hallmark inflammatory responses with Tat-induced pathological alterations in ECs and HBMVECs. The IL6/JAK/STAT3 signaling pathway, in which the cytokine IL-6 activates Janus kinases (JAKs) and subsequently the transcription factor STAT3, represents a critical hallmark of HIV infection (Lin et al., 2021). Rapid activation of inflammation is an essential acute antiviral response to the HIV-1 infection in brain microglia in HIV infected individuals (Walsh et al., 2014). Thus, contrary to the prevailing view that early HIV infection triggers brain inflammation primarily through microglia and astrocyte activation (Jia and Brew, 2025, Kong et al., 2024), our findings suggest that Tat-induced IL-6/JAK/STAT3 signaling in ECs may represent an alternative early mechanism by which HIV-1 entry across the BBB promotes endothelial injury and inflammation.

To further investigate the pathways underlying elevated phalloidin staining intensity in HIV-1 Tat-treated ECs, IPA of RNA-Seq data identified distinct canonical pathways activated in mouse ECs and HBMVECs after Tat exposure. For example, actin filament organization is associated with the activation of several pathways, including extracellular matrix organization, integrin signaling, regulation of actin-based motility by Rho, and signaling by Rho family GTPases. Previous studies indicate that Tat protein does not directly activate actin filament organization but instead modulates cytoskeletal dynamics indirectly through its effects on cellular pathways (Matarrese and Malorni, 2005). Indeed, Tat protein interacts with host Rho family GTPases to activate signaling pathways that contribute to viral replication and pathogenesis. By engaging cell surface receptors such as integrins, Tat triggers RhoA activation, which in turn promotes actin polymerization and cytoskeletal remodeling (Krogh et al., 2014, Zhang et al., 2025, Zhong et al., 2010). Furthermore, Tat protein interacts with the actin cytoskeleton by binding to host cell receptors, activating intracellular signaling pathways, and modeling directly actin-binding proteins, ultimately leading to actin remodeling in Tat-treated ECs (Matarrese and Malorni, 2005).

Although both mouse and human ECs are used in this study, HBMVECs represent a specialized type of ECs derived from the human BBB, characterized by functions such as high trans-endothelial electrical resistance essential for barrier integrity, whereas mouse ECs represent a broader category of endothelial cells originating from diverse mouse tissues (Abbott et al., 2010, Kalucka et al., 2020). IPA of RNA-Seq data revealed several enriched canonical pathways in Tat-treated HBMVECs, including deactivated Class A/1 and CREB signaling, and activated NCAM signaling. Previous studies reported that HIV-1 viral proteins disrupt the BBB, leading to neuroinflammation and neuronal damage via suppressing CREB signaling (Shrestha et al., 2022), while CREB signaling can modulate BBB integrity and be disrupted by factor that compromise the BBB (Lee et al., 2010). CREB dysregulation coupling synaptic activity induces long-term changes in neuronal plasticity (Sakamoto et al., 2011). Accordingly, Tat exposure may damage ECs, thereby contributing to the dysregulation of these pathways. Notably, IPA highlighted BDNF as a central gene consistently associated with all enriched canonical pathways in Tat-treated HBMVECs. Tat protein promotes endothelial dysfunction of the BBB (Chandran, Adler, 2025), which interferes with BDNF gene expression and leads to reduce BDNF production in patients with HIV infection (Belloir et al., 2025, Michael et al., 2024, Vitaliano et al., 2022). Taken together with our findings, enhancing BDNF secretion from the brain endothelium may represent a therapeutic strategy to mitigate deficits in neuronal differentiation and maintenance in HIV-infected patients.

In conclusion, the current findings provide novel insights into endothelial cell-dependent mechanisms of HIV-1-induced neuroinflammation. Traditionally, neuroinflammation in HIV infection has been attributed to viral replication and viral proteins crossing the BBB during the early stages of infection. However, our findings suggest an alternative mechanism: HIV-1 viral proteins disrupt endothelial cell function and upregulate pathways related to immune responses, thereby increasing proinflammatory cytokine levels and contributing to neuroinflammation in HAND. Future studies will investigate whether the effects of *in vitro* Tat exposure on mouse cortical ECs can be recapitulated in the inducible Tat transgenic mouse model. This model exhibits cognitive deficits similar to those observed in chronically HIV-infected patients receiving cART and has been widely used to assess the impact of *in vivo* Tat expression on neurological outcomes. A challenge question is whether the Tat-induced candidate genes identified in the current study can serve as potential therapeutic targets. In particular, we are interested in genes that may contribute to the pathophysiology of HAND.

## Supplementary Material

Supplementary Files

This is a list of supplementary files associated with this preprint. Click to download.

• ZHUTatmousehumanEVsMSFINALSuplInfo.docx

## Data Availability

Except for data of RNA-seq and pathway analysis uploaded in NCBI GEO, all data of this study is available on request from the corresponding author, and some additional data can be accessed from the Supplementary Materials
